# MRI-based study of gray matter volume and cortical morphological alterations in patients with rheumatoid arthritis

**DOI:** 10.1515/rir-2026-0021

**Published:** 2026-07-13

**Authors:** Mingqing Xu, Shipin Zang, Yifan Yang, Ru Bai, Shuang Liu, Ruomei Cui, Shu Li, Yuqi Cheng, Jian Xu

**Affiliations:** Department of Rheumatology and Immunology, First Affiliated Hospital of Kunming Medical University, Kunming, Yunnan Province, China; Affiliated Mental Health Center & Hangzhou Seventh People’s Hospital, Zhejiang University School of Medicine, Hangzhou, Zhejiang Province, China

**Keywords:** rheumatoid arthritis, voxel-based morphometry (VBM), surface-based morphometry (SBM), structural magnetic resonance imaging (sMRI)

## Abstract

**Objective:**

To investigate alterations in gray matter volume (GMV) and cortical morphology in patients with rheumatoid arthritis (RA) and examine their associations with clinical characteristics using voxel-based morphometry (VBM) and surface-based morphometry (SBM).

**Methods:**

41 RA patients and 34 age- and sex-matched healthy controls (HCs) underwent high-resolution structural magnetic resonance imaging (sMRI). VBM was used to compare GMV, and SBM was applied to assess gyrification index (GI), cortical thickness (CT), sulcus depth (SD), and fractal dimension (FD). Whole-brain voxel/vertex-wise multiple linear regression analyses evaluated associations between structural indices and DAS28 disease activity scores, Hamilton Depression Scale (HAMD), Hamilton Anxiety Scale (HAMA), Montreal Cognitive Assessment (MoCA), C-reactive protein (CRP), Visual Analogue Scale (VAS), swollen joint count, and tender joint count.

**Results:**

No significant group diferences were found in sex (χ^2^= 0.98, *P* = 0.32) and age (*t* = 0.48, *P* = 0.14). Compared with HCs, RA patients showed reduced GMV in the bilateral putamen (*P_voxel_* = 0.001, *P_cluster_* = 0.05; cluster size > 50), decreased SD in the left hemisphere (118 vertices: 79% left inferior frontal gyrus, opercular part; 16% lateral orbital frontal gyrus; 5% superior frontal gyrus; *P* = 0.0003), decreased GI in the right superior and inferior parietal gyri (65 vertices; *P* = 0.0004), and reduced CT in the left inferior temporal gyrus (93 vertices; *P* < 0.0001). Several structural indices were correlated with clinical variables. In the RA group, HAMA scores correlated positively with CT in the left superior frontal gyrus and both inferior and superior parts of the right middle frontal gyrus (*P* = 0.0001). VAS scores correlated positively with CT in the left fusiform gyrus and entorhinal cortex (*P* = 0.0001). Swollen joint counts correlated positively with CT in the inferior left middle frontal gyrus (*P* = 0.0001) and right inferior parietal lobule (*P* = 0.0004), positively with FD in the left precuneus (*P* < 0.0001), and negatively with FD in the right inferior parietal gyrus (*P* = 0.0001). DAS28 (CRP) correlated positively with GI and negatively with SD in the opercular part of the left inferior frontal gyrus and the right superior temporal gyrus (*P* = 0.0001). HAMD scores correlated negatively with GI and SD in the left inferior parietal gyrus, right supramarginal gyrus, and right precentral gyrus (GI: *P* = 0.0002, *P* < 0.0001, *P* *=* 0.0001; SD: *P* = 0.0002, *P* < 0.0001, *P* = 0.0003). CRP correlated positively with FD in the right postcentral gyrus and right superior parietal gyrus (*P* < 0.0001). MoCA scores correlated negatively with FD in the left superior frontal gyrus (*P* = 0.0001). Tender joint counts correlated negatively with SD in the left inferior parietal gyrus (*P* < 0.0001) and with FD in the opercular part of the left inferior frontal gyrus (*P* < 0.0001).

**Conclusion:**

Through multi-parameter joint analysis, this study systematically depicted the multi-dimensional changes in brain structure of patients with RA for the first time and initially established a framework for their association with disease activity, emotional and cognitive impairment, and inflammatory levels.

## Background

Rheumatoid arthritis (RA) is a chronic systemic autoimmune disease primarily characterized by persistent synovial inflammation. Typical manifestations include cartilage and bone erosion, predominantly involving the small joints of the hands and feet, with progressive, symmetrical inflammation that ultimately results in cartilage destruction, bone erosion, and joint deformity or disability.^[1]^ The global prevalence of RA is approximately 0.5%–1%, with an incidence two to three times higher in women than in men. The mortality rate among patients with RA is significantly higher than that of the general population.^[2]^ Notably, overt central nervous system (CNS) manifestations such as meningitis, vasculitis, or focal neurological deficits are rarely encountered in routine RA practice. However, accumulating evidence indicates that subclinical CNS involvement—manifested as cognitive impairment (CI), depression, anxiety, and fatigue—is considerably more prevalent, affecting up to 70% of patients with RA.^[3, 4, 5]^ A meta-analysis of five observational studies reported an odds ratio of 1.64 (95% CI: 0.92–2.93) for the association between RA and comorbid CI or dementia; although this result did not reach statistical significance, the association became significant following sensitivity analysis.^[6]^ Karin *et al*.^[7]^ demonstrated in a large longitudinal cohort that middle-aged patients with RA had a significantly higher likelihood of developing CI 20 years later compared with patients suffering from other joint diseases. These neuropsychiatric symptoms often parallel or precede joint manifestations, yet remain underrecognized due to the lack of objective biomarkers and the insensitivity of conventional clinical magnetic resonance imaging (MRI) sequences to subtle structural alterations.

MRI, as a noninvasive, highly reproducible, and radiation-free imaging modality, represents an ideal tool for assessing autoimmune rheumatic diseases (ARDs) that often involve the brain.^[8]^ However, in RA patients with cerebral involvement, conventional MRI findings are often nonspecific or even negative, limiting their ability to provide quantitative assessment of neural alterations. Structural brain changes associated with chronic pain, particularly alterations in gray matter volume (GMV) within the prefrontal cortex, may play a critical role in modulating cognitive function.^[9]^ Therefore, investigating GMV and cortical morphological alterations in RA patients has become an important focus of clinical research. In recent years, voxel-based morphometry (VBM) and surface-based morphometry (SBM) have been widely employed in the quantitative analysis of structural MRI data. VBM enables precise estimation of the relative volumes of gray matter, white matter, and cerebrospinal fluid within a standardized spatial framework, thereby facilitating voxel-wise comparisons of gray and white matter volume. SBM, following skull stripping and signal correction, reconstructs the cortical surface through tissue segmentation and spatial normalization, and computes parameters such as gyrification index (GI), cortical thickness (CT), sulcus depth (SD), and fractal dimension (FD) to quantify and identify cortical structural alterations. Thus, multimodal MRI analysis combining VBM and SBM can provide a more comprehensive understanding of brain structural alterations in RA. We hypothesized that patients with RA would exhibit GMV loss and cortical morphological changes, which may be associated with specific clinical characteristics. This study aimed to provide novel insights into the structural brain alterations associated with RA through comprehensive MRI-based morphometric analysis.

## Methods

### Participants

According to the predefined inclusion and exclusion criteria, patients diagnosed with RA who attended the Department of Rheumatology and Immunology, The First Affiliated Hospital of Kunming Medical University, between 2021 and 2024, and healthy controls (HCs) recruited during the same period were enrolled in this study.

### Inclusion and Exclusion Criteria

Inclusion criteria for patients with RA group were as follows: (1) Age between 18 and 60 years, with no restriction on sex; (2) Meet the 2010 American College of Rheumatology/European League Against Rheumatism (ACR/EULAR) classification criteria for RA; (3) Right-handedness; (4) Voluntary participation with written informed consent obtained; (5) Received treatment with conventional synthetic disease-modifying antirheumatic drugs (csDMARDs; *e.g*., methotrexate, leflunomide, sulfasalazine, iguratimod, hydroxychloroquine) for ≥ 8 weeks with a stable dose for ≥ 4 weeks, or with biologic DMARDs (bDMARDs; TNF-α or interleukin-6 receptor inhibitors) or targeted synthetic DMARDs (tsDMARDs; JAK inhibitors) for ≥ 12 weeks with a stable dose for ≥ 4 weeks; (6) If taking glucocorticoids at screening, maintained a stable dose (prednisone-equivalent ≤ 10 mg/day) for at least 4 weeks prior to MRI scanning.

### Inclusion Criteria for HCs

(1) Age between 18 and 60 years, with no restriction on sex; (2) Right-handedness; (3) Absence of significant anxiety or depression as assessed by the Hamilton Anxiety Scale (HAMA) and Hamilton Depression Scale (HAMD), and absence of CI as determined by the Montreal Cognitive Assessment (MoCA); (4) Provided voluntary participation with signed informed consent. The exclusion criteria applicable to both groups were as follows: (1) Presence of any other autoimmune rheumatic disease; (2) Presence of any other intracranial pathology, major psychiatric disorder, or neurological disease; (3) Family history of neurological or psychiatric disorders; (4) Insufficient educational background to complete neuropsychological assessments; (5) Inability to undergo MRI examination for any reason; (6) Contraindications to MRI scanning, including cardiac pacemakers, metallic braces, or dental prostheses; (7) History of significant head trauma or brain surgery; (8) History of cerebrovascular events, including transient ischemic attack, cerebral infarction, or intracerebral hemorrhage; (9) Individuals with a history of alcoholism, drug abuse, or treatment with antidepressant or antipsychotic medications; (10) Patients with severe organ failure, such as hepatic, renal, or cardiac dysfunction; (11) Claustrophobia or any other condition contraindicating MRI scanning; (12) Pregnancy or lactation; (13) Use of anti-CD20 monoclonal antibody therapy within the previous six months; Any investigational drug or other unmentioned medication within < 5 half-lives; (14) Current or prior malignancy, uncontrolled systemic comorbidities, lymphoproliferative disease, or any acute or chronic infection within six months preceding MRI; (15) Any other medical or psychological condition that could interfere with study completion.

### Data Collection

#### Demographic and Clinical Information

Including sex, age, ethnicity, and years of education, disease duration, medication history, Disease Activity Score in 28 joints (DAS28), visual analogue scale (VAS) score, tender and swollen joint counts, and the presence or absence of large-joint involvement.

#### Laboratory Examinations

For patients with RA, peripheral venous blood samples were obtained from the cubital vein after overnight fasting. Samples were sent to the clinical laboratory for measurement of erythrocyte sedimentation rate (ESR), C-reactive protein (CRP), and autoantibodies, including antinuclear antibody (ANA) and anti–cyclic citrullinated peptide (anti-CCP) antibody.

#### RA Disease Activity Assessment

RA disease activity was evaluated using the DAS28 (ESR/ CRP) scoring system.^[10]^ Based on the total DAS28 score, disease activity was classified as remission (≤ 2.6), low (> 2.6, ≤ 3.2), moderate (> 3.2, ≤ 5.1), or high (> 5.1).

#### Pain Assessment

Pain intensity was assessed using the VAS,^[11]^ ranging from 0 to 10, where 0 indicates no pain, 1–3 mild pain, 4–6 moderate pain, 7–9 severe pain, and 10 the most severe pain.

#### Cognitive and Psychological Assessment

On the day of MRI scanning, all participants were evaluated by a psychiatrist using the HAMD-24 items and HAMA-14 items^[12]^ for depressive and anxiety symptoms. These two scales are widely used in clinical and research settings as self-reporting scales. Higher scores indicate more severe symptoms. In this study, we reported the continuous total scores for correlation analysis. Additionally, to describe the characteristics of the cohort, we referred to the commonly used research cutoff values (HAMD total score < 8 points, normal: 8–20 points, possible dysthymia: 20–35 points, definite dysthymia; > 35 points, severe depression; HAMA result determination: total score ≥ 29 points, may be severe anxiety; ≥ 21 points, definitely has significant anxiety; ≥ 14 points, definitely has anxiety; over 7 points, may have anxiety; if less than 7 points, there are no anxiety symptoms.) to classify the severity of symptoms. Cognitive functions were evaluated using the MoCA scale.^[13]^ This scale covers multiple cognitive domains such as attention, executive function, memory, language, visuospatial ability, abstract thinking, calculation, and orientation. The total score is 30 points, and a score of less than 26 is typically used as a cutoff value for screening cognitive dysfunction.

#### MRI Data Acquisition

All participants underwent cranial MRI on a GE Discovery 750w 3.0T scanner equipped with a 24-channel head coil, using a magnetization-prepared rapid acquisition gradient echo (MPRAGE) sequence. All MRI scans were performed by an experienced neuroradiologist to ensure image quality consistency. The scanning parameters were repetition time (TR) = 8.6 ms; echo time (TE) = 3.2 ms; inversion time (TI) = 450 ms; slice thickness = 1 mm with no interslice gap; matrix = 256 × 256; flip angle= 12°; and 240 contiguous slices covering the entire brain.

#### MRI Data Preprocessing

MRI data preprocessing was conducted using the Statistical Parametric Mapping (SPM12) software and Computational Anatomy Toolbox (CAT12) (http://www.neuro.uni-jena.de/CAT/) on the MATLAB platform (MathWorks, Natick, MA, USA). These toolboxes were primarily used for detailed processing of cortical surface area, thickness, volume, and curvature. The VBM processing includes three steps: (1) Tissue segmentation, adjusting the origin position of the image, and then performing tissue segmentation, dividing the brain tissue into three parts: white matter, gray matter, and cerebrospinal fluid, to generate different brain region images; (2) Spatial standardization was carried out using the spatial template of the Montreal Neurological Institute (MNI), and the brain region results obtained during the analysis were used as MNI coordinates; (3) Smoothing: An 8-mm full width at half maximum (FWHM) Gaussian kernel was applied to smooth the gray matter images, thereby improving signal-to-noise ratio and reducing registration errors introduced during spatial normalization to enhance the robustness of subsequent statistical analyses.

#### SBM Data Processing

Following the CAT12 manual and standard workflow, all SBM indices were computed in each participant’s native space. CT was calculated using the projection-based thickness (PBT) approach. After tissue segmentation, a white matter distance map was generated by estimating the distance between each gray matter voxel and the inner cortical boundary. The outer cortical boundary values derived from the white matter distance map were projected back onto the inner boundary to generate a CT map. Subsequently, a central cortical surface was generated at the 50% position between the white matter distance map and the CT map. For central cortical surface extraction, default settings were applied, and topological defects were corrected using spherical harmonic–based topology correction. The GI was computed using spherical harmonic reconstruction, with vertex-wise GI maps derived through the absolute mean curvature method. A central surface mesh was then reconstructed, representing both the gray matter–cerebrospinal fluid interface and the gray matter–white matter boundary. The absolute mean curvature was computed as the average curvature within a 3-mm neighborhood around each vertex, yielding the local absolute mean curvature for the central surface. The FD reflects the complexity of cortical folding patterns. FD was estimated *via* spherical harmonic reconstruction and calculated as the slope of the logarithmic plot of surface area against the maximum l-value, representing the frequency bandwidth used in surface shape reconstruction. SD, quantifying the cortical sulcal depth, was calculated as the Euclidean distance between the central cortical surface and its convex hull, based on spherical harmonic reconstruction. After generating individual morphometric maps of CT, FD, GI, and SD, the cortical surface was resampled into a standardized coordinate system, followed by Gaussian smoothing. CT maps were smoothed with a Gaussian kernel of 15 mm FWHM, whereas FD, GI, and SD maps were smoothed with a 20-mm FWHM Gaussian kernel. The normalized and smoothed morphometric data were subsequently subjected to statistical analysis.

### Statistical Analysis

Demographic and clinical data were analyzed using SPSS software (version 24.0; IBM Corp., Armonk, NY, USA). Quantitative variables with normal distribution were expressed as mean ± standard deviation (SD) and compared using independent-sample *t* tests or one-way analysis of variance. Non-normally distributed variables were expressed as median (M) (Q1, Q3) and compared using the Mann–Whitney *U* test or Kruskal-Wallis *H* test. Categorical variables were analyzed using *chi-square* (*χ*^2^) tests or Fisher’s exact test. A two-tailed *P* value < 0.05 was considered statistically significant.

VBM analysis: Two-sample *t* tests or one-way analysis of covariance were conducted with age, sex, years of education and total intracranial volume (TIV) as covariates to compare GMV between RA patients and HCs or among different RA patient groups with different disease activity levels and healthy controls. Results were corrected for multiple comparisons using the Gaussian Random Field (GRF) method (*P*_voxel_ = 0.001, *P*_cluster_ = 0.05; cluster size > 50).

SBM analysis: Two sample *t* tests or one-way analysis of covariance were performed with age, sex and years of education as a covariate to evaluate group differences in CT, GI, FD, and SD. The significance threshold was set at *P* < 0.001 (uncorrected), and clusters size > 50 vertices.

Multiple linear regression analysis of structural indices and clinical measures at the whole-brain voxel/vertex level was performed. Structural index maps were entered as dependent variables into the SPM12 multiple linear regression model, with clinical variables as the main independent variables, and age, sex, and years of education included simultaneously as covariates in the design matrix. The statistical significance threshold was set at *P* < 0.001 with a cluster size > 50 voxels/vertices. Statistical parametric maps were generated to reflect the correlations between structural indices and clinical measures after controlling for age, sex, and education.

## Results

### Demographic and Clinical Characteristics

A total of 41 patients with RA and 34 HCs were included in the final analysis. No significant differences in age or sex were observed between the two groups confirming demographic comparability. The RA group showed higher HAMA (*P* = 0.005) and HAMD (*P* < 0.001) scores and lower MoCA (*P* = 0.004) scores compared with the HCs group (Table 1). Based on the critical values of the quantitative scale (HAMA ≥ 7 points, HAMD ≥ 8 points, MoCA < 26 points), among 41 RA patients, 14 patients (34.15%) had anxiety symptoms, 12 patients (29.27%) had depressive symptoms, and 17 patients (41.46%) had cognition impairment.

**Table 1:** Demographic and partial clinical data of RA and HCs groups

Variable	RA Group (*n* = 41)	HCs Group (*n* = 34)	*χ^2^/ t / Z*	*P*-value
Sex				
Male	8	10	0.98	0.32
Female	33	24		
Age (years)	36.5 ± 8.23	33.71 ± 8.10	0.48	0.14
Disease duration (months)	36.50 (12.00, 120.00)			
Years of education (years)	15.00 (6.00, 19.00)	16.00 (16.00, 16.00)	-1.180	0.238
HAMA	5.00 (3.00, 7.00)	3.00 (2.00, 4.00)	-2.777	0.005
HAMD	6.00 (3.00, 8.00)	2.00 (1.00, 4.00)	-3.463	<0.001
MoCA	27.00 (23.00, 28.00)	28.00 (27.00, 29.00)	-2.920	0.004
VAS	5.00 (4.00, 7.00)			
DAS28 (CRP)	4.62 (3.39, 5.92)			
DAS28 (ESR)	5.13 (3.70, 6.40)			
ESR (mm/h)	33.00 (17.25, 46.00)			
CRP (mg/L)	12.00 (4.44, 34.82)			

RA: rheumatoid arthritis; HCs: healthy controls; MoCA: Montreal Cognitive Assessment; HAMA: Hamilton Anxiety Scale; HAMD: Hamilton Depression Scale; DAS28: Disease Activity Score-28; VAS: Visual Analogue Scale, ESR: erythrocyte sedimentation rate; CRP: C-reactive protein; SD: standard deviation.

### Between-group VBM Analysis Results

**Figure 1:**
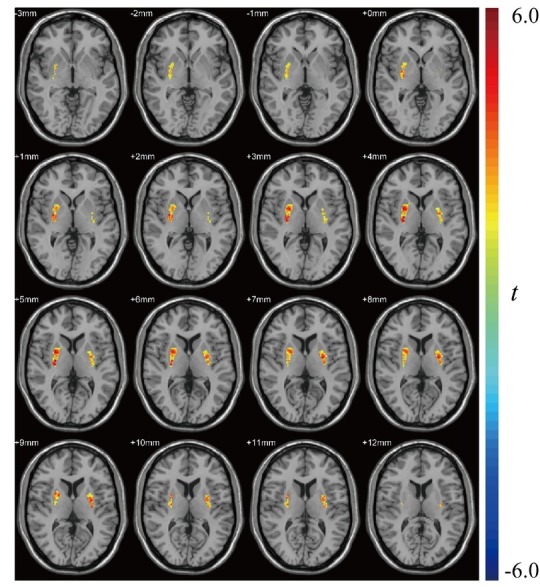
Brain regions with significant differences in GMV between RA (n = 41) and HCs (n = 34). Multiple comparisons were corrected using the GRF method (P_voxel_ = 0.001, P_cluster_ = 0.05; cluster size > 50); The color bar represents t-values (warm colors indicate regions where HCs > RA). GMV: gray matter volume; RA: rheumatoid arthritis; HCs: healthy controls; GRF: Gaussian Random Field.

Compared with HCs, the RA group demonstrated significantly reduced GMV bilaterally in the putamen (Figure 1 and Table 2).

**Table 2:** Brain regions showing significant differences in GMV between RA (n = 41) and HCs (n = 34)

Cluster	Brain Region	Voxels	MNI Coordinates			*P*	*T*
			X	Y	Z		
Cluster 1	Right Putamen	661	30	-16.5	4.5	<0.001	5.95
Cluster 2	Left Putamen	343	-28.5	-15	9	<0.001	5.84

GMV: gray matter volume; RA: Rheumatoid Arthritis; HCs: health control; MNI: Montreal Neurological Institute; X, Y, Z, spatial coordinates in MNI space; Multiple comparisons were corrected using the Gaussian Random Field (GRF) method (*P*_voxel_ = 0.001, *P*_cluster_ = 0.05; cluster size > 50).

### Between-group SBM Analysis Results

**Figure 2:**
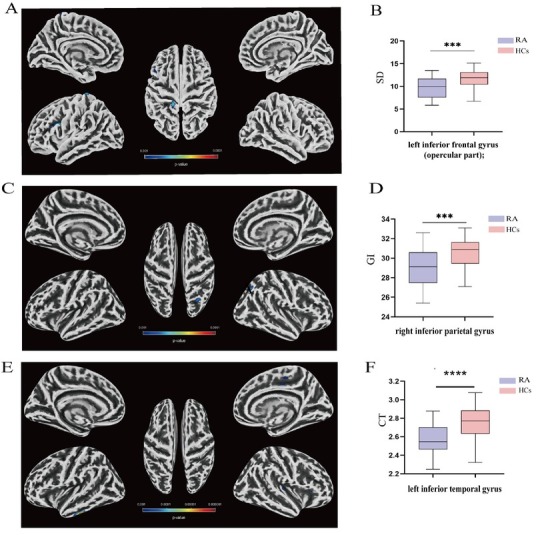
Brain regions showing significant reductions in SD, GI, and CT in the RA group (n = 41) compared with the HCs group (n = 34). (A) Cluster showing significantly decreased SD in RA patients compared with HCs. (B) Comparison of SD values of the left inferior frontal gyrus (opercular part) between the RA and HCs groups. (C) Cluster showing significantly decreased GI in RA patients compared with HCs. (D) Comparison of GI values of the right inferior parietal gyrus between the RA and HCs groups. (E) Cluster showing significantly decreased CT in RA patients compared with HCs. (F) Comparison of CT values of the left inferior temporal gyrus between the RA and HCs groups. Cluster-forming threshold: P < 0.001; cluster-wise threshold: P_value_ = 0.001, *** indicates P < 0.001, **** indicates P < 0.0001. SD: sulcus depth; GI: gyrification index; CT: cortical thickness; RA: rheumatoid arthritis; HCs: healthy controls.

Compared with HCs, the RA group exhibited a cluster in the left hemisphere (118 vertices: 79% located in the left inferior frontal gyrus [opercular part], 16% in the left lateral orbital frontal gyrus, and 5% in the superior portion of the left middle frontal gyrus; *P* = 0.0003) showing decreased SD. In the right hemisphere, a cluster (65 vertices: 50% located in the right superior parietal gyrus and 50% in the right inferior parietal gyrus; *P* = 0.0004) exhibited a significantly reduced GI. Another cluster located in the left hemisphere (93 vertices, entirely located in the left inferior temporal gyrus; *P* < 0.0001) demonstrated decreased CT (Figure 2 and Table 3).

**Table 3:** Brain regions with significant reductions in CT, GI, and SD between RA (n = 41) and HCs (n = 34)

Measure	Cluster	Brain Region Composition	Voxels	MNI Coordinates			*P*	*T*
				X	Y	Z		
SD	lh cluster 1	79% left inferior frontal gyrus (opercular part) 16% left lateral orbital frontal gyrus 5% superior portion of left middle frontal gyrus	118	-37	13	25	0.0003	3.60
GI	rh cluster 1	50% right inferior parietal gyrus 50% right superior parietal gyrus	65	33	-66	41	0.0004	3.51
CT	lh cluster 1	100% left inferior temporal gyrus	93	-54	-30	-31	<0.0001	4.94

CT: cortical thickness; GI: gyrification index; SD: sulcus depth; RA: rheumatoid arthritis; HCs: healthy controls; MNI: Montreal Neurological Institute; X, Y, Z, spatial coordinates in MNI space; lh: left hemisphere; rh: right hemisphere. Voxel-wise *P* < 0.001; cluster size > 50; Brain regions were identified based on the Desikan–Killiany atlas.

### Correlation Analysis

**Figure 3:**
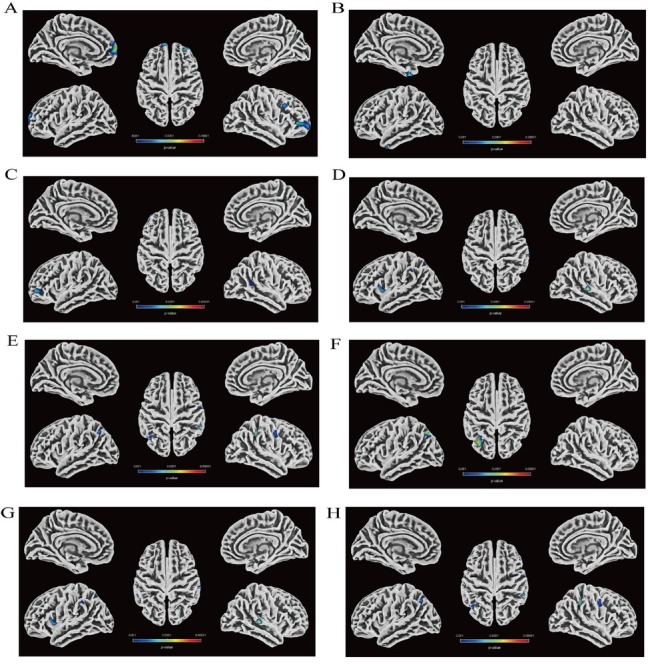
Brain regions showing associations between whole-brain structural measures and clinical variables in patients with RA. (A) Clusters showing positive correlations between CT and HAMA scores. (B) Clusters showing positive correlations between CT and VAS scores. (C) Clusters showing positive correlations between CT and swollen joint counts. (D) Clusters showing positive correlations between GI and DAS28 (CRP) scores. (E) Clusters showing negative correlations between GI and HAMD scores. (F) Clusters showing negative correlations between SD and tender joint counts. (G) Clusters showing negative correlations between SD and DAS28 (CRP) scores. (H) Clusters showing negative correlations between SD and HAMD scores. Cluster-forming threshold: P < 0.001; cluster-wise P = 0.001. CT: cortical thickness; GI: gyrification index; SD: sulcus depth; HAMA: Hamilton Anxiety Scale; VAS: Visual Analogue Scale; DAS28: Disease Activity Score-28; CRP: C-reactive protein; HAMD: Hamilton Depression Scale.

**Figure 4:**
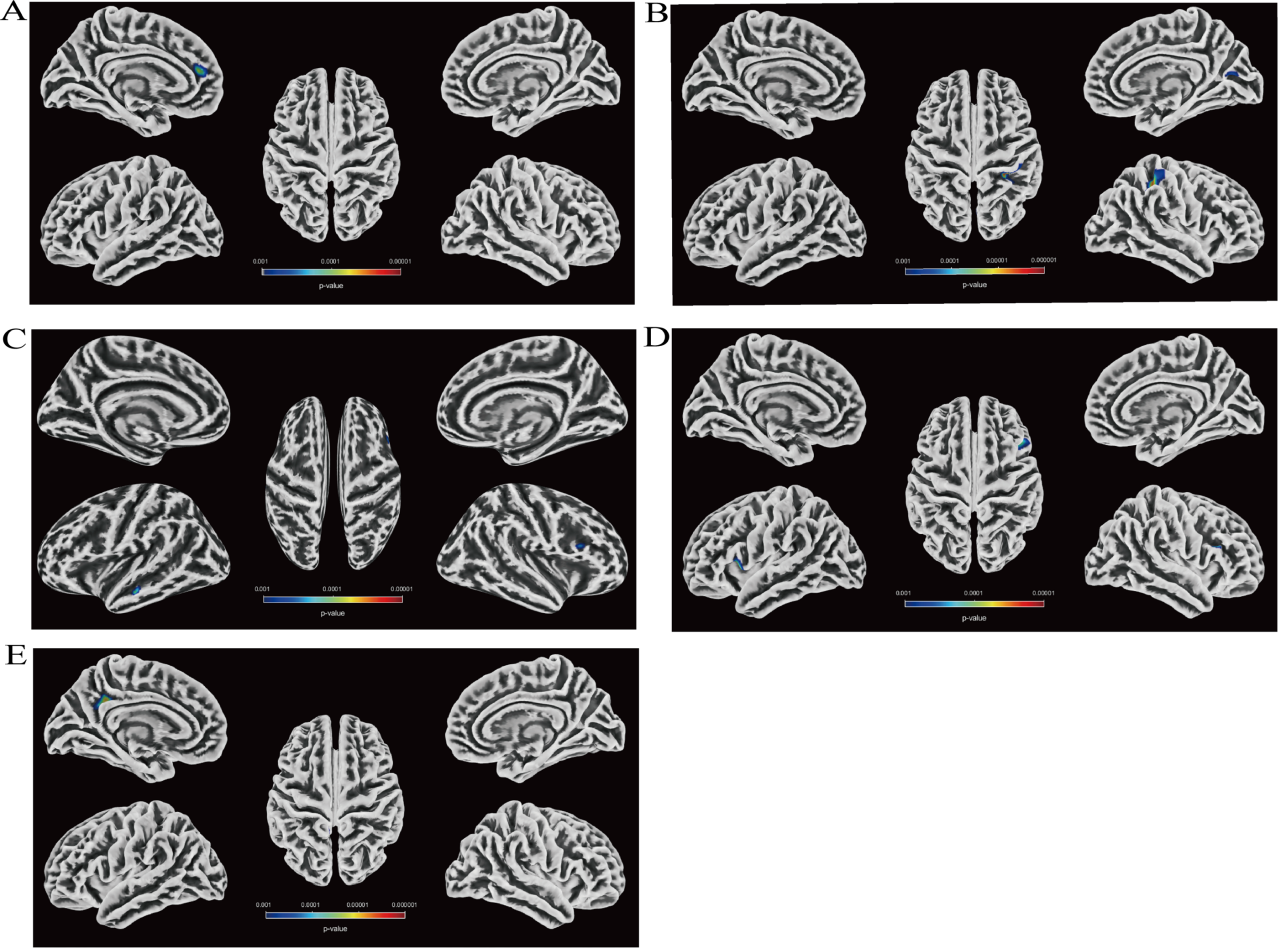
Brain regions showing associations between whole-brain FD and clinical indicators in patients with RA. (A) Clusters showing negative correlations between FD and MoCA scores. (B) Clusters showing positive correlations between FD and CRP levels. (C) Clusters showing negative correlations between FD and swollen joint counts. (D) Clusters showing positive correlations between FD and tender joint counts. (E) Clusters showing positive correlations between FD and swollen joint counts. Cluster-forming threshold: P < 0.001; cluster-wise P = 0.001. FD: fractal dimension; MoCA: Montreal Cognitive Assessment; CRP: C-reactive protein.

Within the RA group, CT in the left superior frontal gyrus_､_the right inferior part of middle frontal gyrus and the right superior part of middle frontal gyrus was positively correlated with HAMA scores (*P* = 0.0001). CT in the left fusiform gyrus and entorhinal cortex showed a significant positive correlation with VAS scores (*P* = 0.0001). CT in the left inferior part of middle frontal gyrus was positively correlated with swollen joint count (*P* = 0.0001), and the right inferior parietal gyrus was positively correlated with swollen joint count (*P* = 0.0004). GI in the left inferior frontal gyrus (opercular part) and right superior temporal gyrus exhibited positive correlations with DAS28 (CRP) (*P* = 0.0001). GI in the left inferior parietal gyrus (*P* = 0.0002), right supramarginal gyrus (*P* < 0.0001) and right precentral gyrus (*P* = 0.0001) were negatively correlated with HAMD scores. SD in the left inferior parietal gyrus was negatively correlated with tender joint count (*P* < 0.0001), while SD in the left inferior frontal gyrus (opercular part) and right superior temporal gyrus showed negative correlations with DAS28 (CRP) scores (*P* = 0.0001). Additionally, SD in the left inferior parietal gyrus (*P* = 0.0002), the right supramarginal gyrus (*P* < 0.0001) and right precentral gyrus (*P* = 0.0003) were negatively correlated with HAMD scores. FD in the left superior frontal gyrus exhibited a negative correlation with MoCA scores (*P* = 0.0001), whereas FD in the right postcentral gyrus and right superior parietal gyrus was positively correlated with CRP levels (*P* < 0.0001). FD in the right inferior parietal gyrus was negatively correlated with swollen joint count (*P* = 0.0001). FD in the left inferior frontal gyrus (opercular part) was positively correlated with tender joint count (*P* < 0.0001), and FD in the left precuneus was positively correlated with swollen joint count (*P* < 0.0001) (Tables 4 and Figures 3 and 4).

**Table 4:** Correlations between whole-brain GI, CT, SD, FD and clinical parameters in RA

morphology index		Hemisphere Brain region	Cluster size	Peak MNI coordinates			*P*	*T*
				X	Y	Z		
CT								
HAMA-	lh cluster	95% superior frontal gyrus 3% frontal pole 2% middle frontal gyrus	149	-8	64	13	0.0001	3.84
	rh cluster 1	91% inferior part of middle frontal gyrus 5% inferior frontal gyrus (triangular part) 4% frontal pole	253	30	51	-3	0.0001	4.62
	rh cluster 2	100% superior part of middle frontal gyrus	63	37	16	36	0.0001	3.71
VAS-	lh cluster	50% fusiform gyrus 38% entorhinal cortex 12% temporal pole	68	-27	2	-42	0.0001	3.72
Swollen joint count –	lh cluster	100% inferior part of middle frontal gyrus	54	-39	51	-1	0.0001	4.43
	rh cluster	100% inferior parietal gyrus	63	47	-50	13	0.0004	3.84
GI								
DAS28 (CRP)-	lh cluster 1	89% inferior frontal gyrus (opercular part) 11% precentral gyrus	109	-47	16	6	0.0001	4.36
	rh cluster	89% superior temporal gyrus 11% superior temporal sulcus	54	68	-28	2	0.0001	4.68
HAMD-	lh cluster 1	100% inferior parietal gyrus	74	-47	-65	43	0.0002	4.08
	rh cluster 1	100% supramarginal gyrus	98	56	-42	40	<0.0001	4.90
	rh cluster 2	100% precentral gyrus	62	53	-2	47	0.0001	4.43
SD								
tender joint count-	lh cluster	93% inferior parietal gyrus 7% superior parietal gyrus	244	-40	-67	48	<0.0001	5.01
DAS28 (CRP)-	lh cluster 1	89% inferior frontal gyrus (opercular part) 11% precentral gyrus	109	-47	16	6	0.0001	4.36
	rh cluster	89% superior temporal gyrus 11% superior temporal sulcus	54	68	-28	2	0.0001	4.68
HAMD-	lh cluster	100% inferior parietal gyrus	74	-47	-65	43	0.0002	4.08
	rh cluster 1	100% supramarginal gyrus	98	56	-42	40	<0.0001	4.01
	rh cluster 2	100% precentral gyrus	49	55	-4	41	0.0003	3.91
FD								
MoCA-	lh cluster	100% superior frontal gyrus	71	-12	43	17	0.0001	4.58
CRP-	rh cluster 1	58% postcentral gyrus 33% superior parietal gyrus 5% inferior parietal gyrus 5% supramarginal gyrus	386	30	-37	51	<0.0001	5.83
Swollen joint count –	rh cluster	100% inferior parietal gyrus	52	51	-49	40	0.0001	4.27
Tender joint count +	lh cluster	100% inferior frontal gyrus (opercular part)	122	-44	11	2	<0.0001	4.94
Swollen joint count +	lh cluster	63% precuneus 37% isthmus cingulate gyrus	126	-15	-47	34	<0.0001	5.44

CT: cortical thickness; GI: gyrification index; SD: sulcus depth; FD: fractal dimension; HAMA: Hamilton Anxiety Scale; HAMD: Hamilton Depression Scale; VAS: Visual Analogue Scale; DAS28: Disease Activity Score-28; CRP: C-reactive protein; MoCA: Montreal Cognitive Assessment; MNI: Montreal Neurological Institute; X, Y, Z, spatial coordinates in MNI space; lh: left hemisphere; rh: right hemisphere. All voxel-wise *P* < 0.001; cluster size > 50; Brain regions were identified based on Desikan–Killiany atlas. + indicates positive correlation, – indicates negative correlation.

### Subgroup Analysis Based on Activity Level

To preliminarily explore the influence of disease activity on brain structure, we conducted an exploratory subgroup analysis. According to DAS28-CRP scores, the 41 RA patients were classified as remission (*n* = 3, 7.3%), mild activity (*n* = 6, 14.6%), moderate activity (*n* = 16, 39.0%), or severe activity (*n* = 16, 39.0%). These four subgroups were then compared with HCs group.

We then performed a one-way ANCOVA for GMV, CT, SD, FD, and GI among these four RA disease activity subgroups and HCs, with age, sex, years of education, and TIV as covariates. Significant diferences in bilateral putamen GMV were detected among the five groups (GRF correction *P*_voxel_ = 0.001, *P*_cluster_ = 0.05). However, post-hoc pairwise comparisons with Bonferroni correction revealed no significant diferences in bilateral putamen GMV between any individual RA disease activity subgroup and HCs. These results are presented in the Supplementary Materials (Supplementary Table 1, Supplementary Figure 1). No significant diferences were found in CT, SD, FD, or GI among the five groups.

## Discussion

RA, as a systemic autoimmune disorder, exerts pathological efects extending far beyond the joints. Among its extra-articular manifestations, involvement of the CNS has garnered growing attention in recent years. In this study, both SBM and VBM were employed to comprehensively assess alterations in GMV and cortical morphology in patients with RA. Our findings revealed widespread abnormalities in GMV and cortical morphometric parameters across multiple brain regions in RA patients. Compared with HCs, patients with RA exhibited SD in the opercular part of the left inferior frontal gyrus, reduced GI in the right superior and inferior parietal gyrus, decreased CT in the left inferior temporal gyrus, and significant reduction in the volume of the bilateral putamen regions, and several brain structural metrics showed correlations with clinical variables. These structural alterations not only support the hypothesis of CNS involvement in RA but also further elucidate its potential neuropathological mechanisms closely associated with disease manifestation and progression.

The RA cohort in this study demonstrated a significant burden of neuropsychiatric comorbidities. A considerable proportion of the patients exhibited symptoms of anxiety (34.15%), depression (29.27%), and cognitive decline (41.46%). This is consistent with the reports that RA, as a systemic inflammatory disease, often involves central impairments such as cognition and emotions.^[4,14,15]^ This further supports the necessity of exploring the brain structural basis of this phenomenon from the perspective of neuroimaging. Compared to the HCs group, the RA group had higher HAMD and HAMA scores, and lower MoCA scores, suggesting poorer cognitive and emotional functioning. A study by Jeon *et al*.^[16]^ indicated that RA patients had a 1.66 times higher risk of depression compared to the control group (adjusted hazard ratio [aHR] = 1.66 [95% CI, 1.61 – 1.71]). Fazel *et al*.^[17]^ proposed that due to chronic pain and long-term inflammation, RA patients often experience symptoms of depression and anxiety. McDowell *et al*.^[18]^ reported that the prevalence of cognitive impairment in RA patients is extremely high and significantly correlated with disease activity, which is consistent with the findings of this study.

Research into the relationship between RA and CI has steadily expanded, yet the mechanisms underlying cognitive decline in RA remain incompletely elucidated. Carotta *et al*.^[19]^ suggested that chronic inflammation in RA may afect the CNS, with such involvement leading to functional impairments manifested as cognitive deficits, emotional disturbances, or behavioral alterations. Notably, Zheng *et al*.^[20]^ reported reduced putaminal volume in RA and proposed that CI may arise, at least partly, from alterations in central neural networks, particularly within thalamic structures. Multiple studies have advanced various hypotheses to explain its potential underlying etiological mechanisms, Among these factors, chronic systemic inflammation has been shown to exert a particularly profound influence on cognitive function. Such pathological mechanisms may induce alterations in GMV within the prefrontal cortex—a key region involved in cognitive regulation, and such changes have been associated with anxiety, depression, and cognitive impairment. This is consistent with the findings of our study.

In the study, VBM analysis revealed significantly reduced GMV in the bilateral putamen of RA patients. As a key component of the basal ganglia, the putamen participates not only in motor control but also in emotion regulation, reward processing, and motivational behavior. Putaminal atrophy, as observed in this study, has also been reported in other disorders such as major depression and Parkinson’s disease. For instance, Lu *et al*.^[21]^ reported that structural abnormalities of the putamen may occur in the early stages of major depressive disorder (MDD), suggesting that the putamen could represent a common target of neurodegenerative alteration across various diseases. In our study, putaminal volume was negatively correlated with HAMD scores, further supporting its potential role in CNS involvement in RA. The spatial distribution of structural brain alterations in RA partially overlaps with the morphological abnormalities observed in depression and schizophrenia, predominantly affecting interconnected networks across the frontal, insular, parietal, and temporal cortices. This cross-disease convergence suggests that diverse systemic disorders may induce widespread neural structural alterations *via* shared inflammatory pathways.

Furthermore, we conducted an exploratory analysis stratifying patients into four disease activity levels to investigate whether GMV alterations in the bilateral putamen varied across the activity spectrum. While the one-way ANCOVA revealed a significant overall group effect among the five groups (GRF-corrected), post-hoc Bonferroni-corrected comparisons failed to detect significant pairwise differences between any RA subgroup and HCs. This absence of post-hoc significance is most likely driven by insufficient statistical power due to markedly unequal and small subgroup sizes (remission: *n* = 3; mild: *n* = 6), rather than a true absence of biological effects. Thus, although our findings hint that disease activity may modulate the magnitude of putaminal GMV reduction, this exploratory subgroup analysis remains underpowered to establish a dose-response relationship between disease activity level and brain structural alterations.

In the present study, FD in the left superior frontal gyrus was negatively correlated with MoCA scores, further indicating that reduced cortical structural complexity in RA patients is associated with CI. FD, a sensitive indicator of cortical folding complexity, reflects the efficiency of local information integration. A reduction in FD typically indicates diminished local integrative capacity, potentially associated with decreased dendritic arborization and reduced synaptic density.^[22]^ Lower FD may therefore reflect impaired local information integration, potentially contributing to deficits in executive function and sustained attention in RA patients. Accordingly, abnormal FD may serve as a potential early neuroimaging biomarker of CNS involvement in RA.

Meanwhile, RA patients showed significantly reduced SD in the opercular portion of the left inferior frontal gyrus. SD serves as an important indicator of cortical folding,^[23]^ and its reduction often suggests impaired local cortical development or remodeling.^[24]^ The opercular part of the inferior frontal gyrus, an essential component of Broca’s area, is involved not only in language processing but also in emotional regulation and integration of pain perception. The insular, a core hub for interoceptive awareness, visceral sensation, and emotional integration,^[25]^ may contribute to heightened pain sensitivity and emotional disturbances in RA when structurally altered. Persistent chronic pain may permit continuous peripheral inflammatory signaling to the CNS, leading to glial activation and central sensitization,^[26]^ thereby amplifying pain perception. In our study, SD of the opercular part of the left inferior frontal gyrus was negatively correlated with DAS28 scores, indicating that higher disease activity was associated with greater cortical structural impairment in this region. This finding further supports the notion of progressive central structural damage driven by chronic inflammation. Therefore, the central alterations observed in RA should not be viewed merely as secondary to chronic pain but rather as shared neural representations of systemic inflammation within the CNS. This perspective transcends the traditional joint-centered paradigm, promoting a more integrative understanding of RA as a systemic disorder affecting both peripheral and central structures.

Moreover, the decreased GI in the right superior and inferior parietal gyri further indicated impairment of higher-order cognitive networks in RA patients. The parietal lobe functions as a critical hub of attention and working memory networks, and its structural simplification may underline the reduced attention and executive function observed in RA patients on MoCA testing. Alterations in parietal GI have been reported in multiple neuropsychiatric disorders, including depression^[27]^ and schizophrenia,^[28]^ underscoring its central role in cognitive regulation. Furthermore, the marked reduction in CT in the left inferior temporal gyrus suggests neuronal loss or synaptic degeneration within brain regions involved in pain perception, memory encoding, and emotional processing. Under chronic inflammatory conditions in RA, peripheral proinflammatory cytokines such as TNF-α and IL-6 may cross the blood–brain barrier,^[29]^ activate microglia, and induce neuroinflammatory cascades,^[30]^ ultimately resulting in cortical atrophy.

Importantly, CT, SD, GI, FD demonstrated significant associations with clinical variables, including DAS28 (CRP), HAMD, HAMA, MoCA, CRP, VAS, swollen joint count, and tender joint count. These correlations suggest that both cortical and subcortical alterations may reflect the cumulative impact of systemic inflammation, pain-related central sensitization, and disease chronicity on the brain. A persistent systemic inflammatory state may directly or indirectly induce neuronal apoptosis and glial activation through cytokine-mediated neurotoxicity, oxidative stress, and mitochondrial dysfunction, resulting in decreased GMV and widespread cortical morphological alterations.^[31]^ Furthermore, chronic pain itself may trigger central sensitization and remodeling of the pain matrix, further aggravating structural brain damage. In addition, long-term exposure to glucocorticoids or DMARDs may also influence brain structural integrity to some extent Collectively, the multi-dimensional abnormalities observed in this study underscore the relevance of cortical and subcortical structural metrics as potential neuroimaging markers for characterizing central involvement in RA.

The extensive changes in brain structure observed in this study can be detected through quantitative magnetic resonance imaging, but they are often asymptomatic in routine clinical practice. This highlights the crucial difference between subclinical central nervous system involvement in RA and the obvious neurological diseases. These changes are likely to represent the neuroanatomical basis for linking systemic inflammation to the common neurological and psychiatric symptoms in rheumatoid arthritis. The significant correlations among the morphological indicators suggest that these brain changes are not random phenomena but are closely related to the disease process. They may be driven by chronic peripheral inflammatory signals crossing the blood-brain barrier, leading to glial activation, neurotoxicity, and ultimately resulting in the loss of synapses and neurons.^[32;33]^ Therefore, these magnetic resonance imaging results should be regarded as early, quantifiable indicators of the vulnerability of the central nervous system in rheumatoid arthritis, reflecting both the downstream effects of persistent systemic pathology and the intrinsic components of the disease’s systemic nature. Their value lies in providing an objective benchmark for future longitudinal studies to determine whether effective anti-inflammatory treatments can slow down this central progression and enhance clinicians’ understanding of active assessment and management of brain health in patients with rheumatoid arthritis.

There are several notable limitations in this study. First, the sample size was relatively small (*n* = 41 for RA patients), potentially limiting the statistical power and generalizability of the findings. Meanwhile, the sample size of patients in the remission stage was too small, preventing an effective comparison between the active stage and the remission stage. Replication in larger, multi-center cohorts is necessary to validate these findings. In particular, a sufficient number of patients in long-term remission should be included to clearly distinguish between the state-of-the-art changes related to disease activity and the inherent, characteristic or cumulative damage of the disease. Second, the cross-sectional design precludes causal inference; we cannot determine whether observed structural alterations precede or follow clinical symptom onset, nor whether they progress or stabilize over time. Third, although we established a demographically matched healthy control group, the absence of patients with other immune-mediated inflammatory diseases (*e.g*., systemic lupus erythematosus) or chronic pain disorders as positive controls limits our ability to determine whether the observed neuroimaging patterns are specific to the pathophysiology of RA or represent generalized consequences of chronic systemic inflammation. Future multicenter studies incorporating such disease control cohorts may help disentangle disease-specific from shared autoimmune-related brain structural signatures. Additionally, integrating multimodal neuroimaging with fluid biomarkers—such as neurofilament light chain (NfL) and glial fibrillary acidic protein (GFAP) —would further elucidate the molecular mechanisms underlying CNS involvement in RA from a multi-omics perspective.

## Conclusion

In summary, this study systematically delineated the pattern of brain structural damage in patients with RA. It also established a preliminary framework linking these structural changes to disease activity, emotional and cognitive disturbances, and systemic inflammation. Cortical structural parameters may serve as early objective neuroimaging biomarkers of cerebral involvement in RA, of-fering a scientific basis for early identification and timely intervention.

## Supplementary Material

Supplementary Material Details

## References

[1] Díaz-González F, Hernández-Hernández MV (2023). Rheumatoid arthritis. Med Clínica Engl Ed.

[2] Finckh A, Gilbert B, Hodkinson B (2022). Global epidemiology of rheumatoid arthritis. Nat Rev Rheumatol.

[3] Katchamart W, Narongroeknawin P, Phutthinart N (2019). Disease activity is associated with cognitive impairment in patients with rheumatoid arthritis. Clin Rheumatol.

[4] Drakes DH, Fawcett EJ, Yick JJJ (2025). Beyond rheumatoid arthritis: a meta-analysis of the prevalence of anxiety and depressive disorders in rheumatoid arthritis. J Psychiatr Res.

[5] Pigłowska-Juhnke A, Stanisławska-Kubiak M, Kalmus P, Waszczak-Jeka M, Samborski W, Mojs E (2025). Cognitive impairment in rheumatoid arthritis: the role of pain, inflammation, and multimorbidity in neuropsychological outcomes. Biomedicines.

[6] Cooper J, Pastorello Y, Slevin M (2023). A meta-analysis investigating the relationship between inflammation in autoimmune disease, elevated CRP, and the risk of dementia. Front Immunol.

[7] Wallin K, Solomon A, Kåreholt I, Tuomilehto J, Soininen H, Kivipelto M (2012). Midlife rheumatoid arthritis increases the risk of cognitive impairment two decades later: a population-based study. J Alzheimers Dis.

[8] Markousis-Mavrogenis G, Koutsogeorgopoulou L, Dimitroulas T (2020). Is There a Brain/Heart Interaction in Rheumatoid Arthritis and Seronegative Spondyloartropathies? A Combined Brain/ Heart Magnetic Resonance Imaging Reveals the Answer. Curr Rheumatol Rep.

[9] Almeida Said F, Betoni TB, Magalhaes V, Nisihara R, Skare TL (2019). Rheumatoid arthritis and cognition dysfunction: lack of association with cumulative glucocorticoid use. Immunopharmacol Immunotoxicol.

[10] Prevoo MLL, Van’T Hof MA, Kuper HH, Van Leeuwen MA, Van De Putte LBA, Van Riel PLCM (1995). Modified disease activity scores that include twenty-eight-joint counts development and validation in a prospective longitudinal study of patients with rheumatoid arthritis: modified disease activity score. Arthritis Rheum.

[11] Åström M, Thet Lwin ZM, Teni FS, Burström K, Berg J (2023). Use of the visual analogue scale for health state valuation: a scoping review. Qual Life Res.

[12] Vindbjerg E, Makransky G, Mortensen EL, Carlsson J (2019). Cross-cultural psychometric properties of the Hamilton depression rating scale. Can J Psychiatry.

[13] Danquah MO, Yan E, Lee JW (2024). The utility of the Montreal cognitive assessment (MoCA) in detecting cognitive impairment in surgical populations–A systematic review and meta-analysis. J Clin Anesth.

[14] Meade T, Joyce C, Perich T (2024). Prevalence, Severity, and Measures of Anxiety in Rheumatoid Arthritis: A Systematic Review. Arthritis Care Res (Hoboken).

[15] Pankowski D, Wytrychiewicz-Pankowska K, Pisula E, Fal AM (2025). Prevalence of neurocognitive impairment in patients with rheumatoid arthritis–a systematic review and meta-analysis. Clin Neuropsychol.

[16] Jeon KH, Han K, Jung J (2024). Rheumatoid arthritis and risk of depression in South Korea. JAMA Netw Open.

[17] Seyedeh D Fazel, Massimo Carollo, Lisanne Tap (2025). Impact of Disease-Modifying Antirheumatic Drugs on Cognitive Function in Older Adults with Rheumatoid Arthritis. Drugs Aging.

[18] McDowell B, Marr C, Holmes C (2022). Prevalence of cognitive impairment in patients with rheumatoid arthritis: a cross sectional study. BMC Psychiatry.

[19] Carbotte RM, Denburg SD, Denburg JA (1995). Review cognitive deficit associated with rheumatic diseases: neuropsychological perspectives. Arthritis Rheum.

[20] Zheng Y, Xie L, Huang Z (2024). Functional dysconnectivity and microstructural impairment of the cortico-thalamo-cortical network in women with rheumatoid arthritis: a multimodal MRI study. Heliyon.

[21] Lu Y, Liang H, Han D (2016). The volumetric and shape changes of the putamen and thalamus in first episode, untreated major depressive disorder. NeuroImage Clin.

[22] Beniaguev D, Segev I, London M. (2021). Single cortical neurons as deep artificial neural networks. Neuron..

[23] Im K, Lee J-M, Won Seo S, Hyung Kim S, Kim SI, Na DL (2008). Sulcal morphology changes and their relationship with cortical thickness and gyral white matter volume in mild cognitive impairment and Alzheimer’s disease. NeuroImage.

[24] Joza S, Delva A, Tremblay C (2025). Distinct brain atrophy progression subtypes underlie phenoconversion in isolated REM sleep behaviour disorder. EBioMedicine.

[25] Simone L, Caruana F, Borra E (2025). Anatomo-functional organization of insular networks: From sensory integration to behavioral control. Prog Neurobiol.

[26] Nijs J, George SZ, Clauw DJ (2021). Central sensitisation in chronic pain conditions: latest discoveries and their potential for precision medicine. Lancet Rheumatol.

[27] Peng D, Shi F, Li G (2015). Surface vulnerability of cerebral cortex to major depressive disorder. PLoS One.

[28] Nesvåg R, Schaer M, Haukvik UK (2014). Reduced brain cortical folding in schizophrenia revealed in two independent samples. Schizophr Res.

[29] William A Banks (2005). Blood-brain barrier transport of cytokines: a mechanism for neuropathology. Curr Pharm Des.

[30] Galea I (2021). The blood-brain barrier in systemic infection and inflammation. Cell Mol Immunol.

[31] Liu T, Kong X, Qiao J, Wei J (2025). Decoding Parkinson’s Disease: The interplay of cell death pathways, oxidative stress, and therapeutic innovations. Redox Biol.

[32] Schrepf A, Kaplan CM, Ichesco E (2018). A multi-modal MRI study of the central response to inflammation in rheumatoid arthritis. Nat Commun.

[33] Lai P-H, Wang T-H, Zhang NY, Wu KC, Yao CJ, Lin C-J (2021). Changes of blood-brain-barrier function and transfer of amyloid beta in rats with collagen-induced arthritis. J Neuroinflammation.

