# Supplementary material for: MRI-based study of gray matter volume and cortical morphological alterations in patients with rheumatoid arthritis
Source: Rheumatol Immunol Res. 2026 Jul 13;7(2):104–16. doi: 10.1515/rir-2026-0021 (PMC13358953; doi:10.1515/rir-2026-0021)
Supplement: Supplementary file 1 — Supplementary Material Details [file rir-2026-0021_sm.pdf]

# Supplementary materials

**Supplementary Table 1. Brain regions showing significant group differences in gray matter volume among patients with RA (remission, mild, moderate, and severe activity) and HCs**

| Cluster | Brain Region | Voxels | MNI Coordinates | | | *P* | *F* |
|---|---|---|---|---|---|---|---|
| | | | X | Y | Z | | |
| Cluster 1 | Right Putamen | 918 | 28.5 | -10.5 | 6 | < 0.001 | 9.78 |
| Cluster 2 | Left Putamen | 775 | -25.5 | -4.5 | 7.5 | < 0.001 | 10.44 |

RA: Rheumatoid Arthritis; HCs: health control; corrected for multiple comparisons using the Gaussian Random Field (GRF) method ($P_{voxel}$ = 0.001, $P_{cluster}$ = 0.05). RA-remission (*n* = 3), RA-mild activity (*n* = 6), RA-moderate activity (*n* = 16), RA-severe activity Group (*n* = 16) and HCs (*n* = 34). MNI, Montreal Neurological Institute; X, Y, Z, spatial coordinates in MNI space.

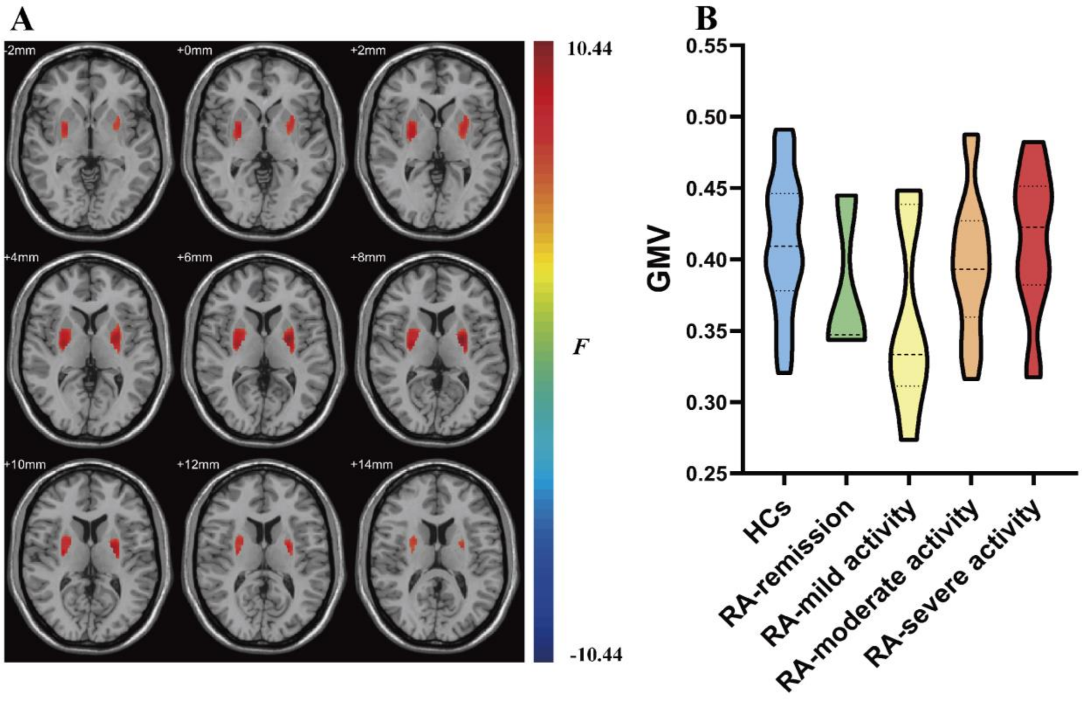


**Supplementary Figure 1. Gray Matter Volume Differences Stratified by Rheumatoid Arthritis Disease Activity: Significant Brain Regions and Post-hoc Comparisons Across Activity Levels and HCs.** GMV: gray matter volume; RA: rheumatoid arthritis; HCs: healthy controls; GRF: Gaussian Random Field. (A) Brain regions showing significant group differences in GMV among patients with RA (remission, mild, moderate, and severe activity) and HCs. GRF correction $P_{\text{voxel}} = 0.001$, $P_{\text{cluster}} = 0.05$; (B) Post-hoc analysis with Bonferroni correction revealed no significant differences in bilateral putamen GMV among the RA disease activity subgroups and HCs. The color bar represents *F*-values.
